# 

**Published:** 1911-11-04

**Authors:** 


### BOOKS RECEIVED.

Henry Frowde and Hodder and Stoughton.
"Manual of Surgery." By Thomson and Miles.

Macmillan and Co., Ltd.
"Conduct and Its Disorders." By Charles A. Mercier.

P. S. King and Son.
"National Conference on the Prevention of Destitution."

C. Richter and Co.
"The Transfusion of Life." By Dr. L. H. Joizet.

### Buildings Contemplated and in Progress.

DEVIZES.—The foundation-stone of the Edward VII. memorial wards and the Grant Meek children's ward was laid on October 18 at the Devizes Hospital. Freemasons will be interested to know that the ceremony was conducted in due form by the Earl of Radnor. An interesting account of a unique ceremony may be read in the *Wiltshire Advertiser* of October 19.

LIVERPOOL.—Messrs. Haigh and Thompson, of Liverpool, are architects for a new operating theatre, etc., to be erected at Brownlow Hill Workhouse. Tenders are invited and particulars may be had from the architects.

STAINES.—The Staines Joint Hospital Committee have had plans prepared by the local surveyor for an isolation hospital to be erected between Staines and Ashford. Tenders are invited and are to be deposited with the Clerk before November 10.

### Appointments.

JOHN PRESCOTT HEDLEY, M.A., M.B., F.R.C.S., M.R.C.P,, has been appointed a physician to out-patients to the British Lying-in Hospital.

ROYAL FREE HOSPITAL: House physician (male), Mr. J. H. Cumming, M.R.C.S., L.R.C.P.; house physician (female), Miss M. Scott, M.B., B.S.; house surgeon (male), Mr. R. E. Porter, M.R.C.S., L.R.C.P.; house surgeon (female), Miss M. D. Thackrah, M.B., B.S.; assistant anæsthetist, Miss M. Rawlins, M.B., B.S.

THE following appointments have been made at the Hampstead General and North-West London Hospital:— W. Stephen Fenwick, M.S., M.B., B.Sc.Lond., F.R.C.S., Eng., Surgeon to out-patients; A. Atkins Greenwood, M.B., B.S.Lond., M.R.C.S., L.R.C.P., Medical Officer for *x*-ray and electrical department; A. J. Shinnie, M.B., Ch.B., House Surgeon; A. M. Stuart, M.R.C.S., L.R.C.P., House Physician.

The following appointments have been confirmed at the monthly meeting of the Manchester Royal Infirmary: Mr. Howard Buck, F.R.C.S., Eng., to be Resident Surgical Officer; Mr. W. H. Hey, F.R.C.S. Eng., to be Assistant Surgical Officer (Out-patient Department); Mr. Graham M. Benton, M.B., Ch.B. Vict., to be Sixth Anæsthetist; Mr. Cecil Hibbert, F.R.C.S. Edin., to be Central Branch Medical Officer (re-appointment); Mr. C. G. Brentnall, M.B., Ch.B. Vict., to be House Physician; Mr. John Morley, F.R.C.S. Eng., to be Surgical Registrar.

### The Florence Nightingale Memorial.

QUEEN ALEXANDRA'S SPLENDID GIFT.

Below we publish the first list of contributions, from which it will be seen that about £2,000 is still needed to complete this memorial. The contributions we announce to-day have been sent in response to the article in The Hospital of October 21. It will, we are sure, strike a tender chord in the hearts of many English people, everywhere, to know that her Majesty Queen Alexandra has written a letter about "our great and good Miss Nightingale's Memorial," and has sent £100 "to perpetuate the memory of the nation's greatest benefactress of suffering humanity."

We asked for money for a statue knowing full well that statues are properly objectionable to many people. But in this case we do not see any way whereby we can so effectually perpetuate the memory and the example of Florence Nightingale for coming generations as by a statue of infinite beauty and best workmanship placed, say, in Waterloo Place, opposite the Crimean Memorial. There is, however, another object to which contributions may be sent—namely, the provision of annuities for trained nurses who have been unable to provide adequately for their old age or infirmity.

It will be observed that sums have been sent in from 1s. to £100. The list now open is therefore within the means of everyone, and all must surely realise the immense gratitude everybody owes to the late Florence Nightingale. Contributions should be addressed to the Editor of The Hospital, 29 Southampton Street, Strand, and should reach the office by the first post Wednesday morning to be acknowledged in the same week's issue.

The following contributions have been received; they are for a statue, except otherwise indicated:—

| | £ | s. | d. |
|---|---|---|---|
| Her Majesty Queen Alexandra ... ... | £100 | 0 | 0 |
| The Duchess of Somerset (promise to collect) | 50 | 0 | 0 |
| Anonymous ... ... ... ... ... | 50 | 0 | 0 |
| Sir Alexander Henderson, Bart. ... ... | 50 | 0 | 0 |
| The Hospital ... ... ... ... ... | 50 | 0 | 0 |
| Sir Henry Burdett, K.C.B. ... ... ... | 25 | 0 | 0 |
| Frederick Cox, Esq., J.P. (for annuities for nurses) ... ... ... ... ... ... | 25 | 0 | 0 |
| George Courtauld, Esq., J.P. ... ... ... | 25 | 0 | 0 |
| Robert Nivison, Esq. ... ... ... ... | 25 | 0 | 0 |
| Lady Wernher ... ... ... ... ... | 25 | 0 | 0 |
| Sir Thomas Lipton ... ... ... ... | 15 | 0 | 0 |
| Alfred de Rothschild, Esq. ... ... ... | 10 | 10 | 0 |
| Leopold de Rothschild, Esq. ... ... ... | 10 | 10 | 0 |
| Leonard L. Cohen, Esq. ... ... ... | 5 | 5 | 0 |
| Frederick Green, Esq., J.P. ... ... ... | 5 | 5 | 0 |
| Robert Gordon, Esq. ... ... ... ... | 5 | 0 | 0 |
| Paul Waterhouse, Esq., F.R.I.B.A. ... ... | 5 | 5 | 0 |
| Miss Davies ... ... ... ... ... | 2 | 2 | 0 |
| Dora Countess of Chesterfield ... ... ... | 1 | 1 | 0 |
| Mrs. Eleanor C. Smyth ... ... ... ... | 1 | 1 | 0 |
| C. F. Ware ... ... ... ... ... | 0 | 5 | 0 |
| F. C. ... ... ... ... ... ... | 0 | 2 | 6 |
| Miss C. F. Fellows ... ... ... ... | 0 | 2 | 6 |
| Miss Ada Wilson ... ... ... ... ... | 0 | 2 | 6 |
| "A Scottish Highlander" ... ... ... | 0 | 1 | 0 |

### The Week's Novelties.

### THE WATERLEAP PATENT FLANNEL-WASHER.

THOSE interested in laundry work would do well to examine the merits of this machine, which appears to be eminently suitable for hospital laundries. Messrs. R. G. Whitaker, Ltd., Academy Works, Fanshaw Street, Hoxton, can show the "Waterleap" washer working in some of the best laundries in London, and invite any reader who may be interested to inspect the machine in actual operation. It washes by water action only, and is particularly suited for washing of flannels, where it would effect a large economy in blankets, flannel jackets, etc., owing to the usual wear and tear and shrinkage of the ordinary machine being done away with.

### "VELURE."

IN our previous reference to the Japan paint known as "Velure," and manufactured by C. Chancellor & Co., 13 Clerkenwell Road, E.C., we inadvertently implied that this varnish paint is stiffer to wash than other paints. We are glad to learn that this is not the case. "Velure" is not stiff to wash, but can be cleaned by merely rubbing down with a wet cloth where other paints require the application of soap and soda and a scrubbing-brush.

AT the last House Committee meeting of the Bristol General Hospital, Dr. George Parker was re-elected physician for a period of ten years; Dr. Newman Nield was re-elected assistant physician, and Dr. F. C. Bergin was appointed skiagraphist.

### The Hospital.

### *SUBSCRIPTION FORM.*

RATES OF SUBSCRIPTION (payable in advance).

UNITED KINGDOM.

| | | |
|---|---|---|
| Three Months (including Postage) | ... | 2s. 0d |
| Six Months | do. | 4s. 0d. |
| Twelve Months | do. | 6s. 6d. |

FOREIGN AND COLONIAL.

| | | |
|---|---|---|
| Three Months (including Postage) | ... | 2s. 6d. |
| Six Months | do. | 5s. 6d. |
| Twelve Months | do. | 8s. 8d. |

*Please send me* "THE HOSPITAL" *for*..........................*months, commencing with your issue of*.........................................., *for which please find enclosed*........................

*Name*..........................................................................

*Address*.......................................................................

..........................................................................

*Date*......................................

This form may be sent to any Newsvendor, Railway Bookstall, or to THE MANAGER, "THE HOSPITAL," 28/9, Southampton Street, Strand, W.C.

